# Elevated angiography-derived microvascular resistance and HbA1c levels jointly predict adverse outcomes in patients with diabetic STEMI: a multicenter retrospective cohort study

**DOI:** 10.3389/fendo.2026.1756159

**Published:** 2026-06-22

**Authors:** Shiyi Gao, Yu Wang, Jun Wang, Qiang Zeng, Zengwei Cheng, Zichen Han, Hongju Wang, Miaonan Li, Sigan Hu

**Affiliations:** 1 Department of Cardiovascular Medicine, The First Affiliated Hospital of Bengbu Medical University, Bengbu, China; 2 Department of Gynecology, The First Affiliated Hospital of Bengbu Medical University, Bengbu, China; 3 Department of Cardiovascular Medicine, Wuhe County People’s Hospital, Bengbu, China; 4 Department of Cardiovascular Medicine, Suzhou No. 1 People’s Hospital, Suzhou, China

**Keywords:** angiography-derived microvascular resistance, hemoglobin A1c, major adverse cardiac and cerebral events (MACCEs), ST-segment elevation myocardial infarction, type 2 diabetes mellitus

## Abstract

**Introduction:**

Research on coronary microcirculatory function in type 2 diabetes mellitus (T2DM) patients with ST-segment elevation myocardial infarction (STEMI) remains limited.

**Methods:**

Patients diagnosed with new-onset STEMI between January 2022 and December 2023 were enrolled. All underwent culprit vessel revascularization, with simultaneous blood sampling and angiography-derived microvascular resistance (AMR) assessment in the reperfused vessel. The primary endpoint was the incidence of major adverse cardiovascular and cerebrovascular events (MACCEs).

**Results:**

A total of 545 patients were divided into four groups based on HbA1c and AMR levels. After adjustment for covariates using a multivariable Cox proportional hazards model, HbA1c (HR = 1.403, 95% CI: 1.131-1.740), AMR (HR = 1.213, 95% CI: 1.105-1.520), and NT-proBNP (HR = 1.244, 95% CI: 1.1053-2.868) remained independently associated with MACCEs. Patients with HbA1c ≥6.5% and AMR ≥250.5 mmHg·s/m exhibited the highest risk of MACCEs (HR = 6.903, 95% CI:3.260-14.618, P < 0.001). Restricted cubic spline analysis revealed nonlinear relationships between MACCEs and both HbA1c and AMR levels. RCS analysis indicated nonlinear relationships between MACCEs incidence and both HbA1c and AMR levels.

**Conclusion:**

This study demonstrates that an elevated AMR following PCI independently predicts MACCEs in patients with STEMI and T2DM. Combined evaluation of AMR and HbA1c enhances risk stratification for MACCEs. Assessment of coronary microcirculatory function and sustained optimization of glycemic control may contribute to improved long-term clinical outcomes in this population.

## 1 Introduction

A continuous increase in the mortality rate associated with acute ST-segment elevation myocardial infarction (STEMI) is being observed globally (1, 2). Although percutaneous coronary intervention (PCI) has been shown to significantly improve patient survival rates, emerging evidence indicates that certain individuals remain at risk for major adverse cardiovascular and cerebrovascular events (MACCEs) subsequent to the procedure (3–5).

Among the diverse risk factors, type 2 diabetes mellitus (T2DM) stands out as a well-established contributing factor (6–9). Clinical evidence indicates that approximately one-third of individuals with T2DM exhibit an elevated risk of STEMI and are more likely to experience adverse clinical outcomes (10–14). This unfavorable prognosis is intricately associated with coronary microvascular dysfunction (CMD) (15). In recent years, the advancement of quantitative flow ratio (QFR) technology has offered a novel approach for the assessment of CMD. QFR has emerged as a promising alternative to fractional flow reserve (FFR) (16–19). Moreover, the angiography-derived microvascular resistance (AMR) index derived from this technology can quantify microcirculation resistance (20).

Diabetic microangiopathy is one of the most prevalent comorbidities associated with T2DM (21). Studies have demonstrated that in diabetic patients without evident epicardial stenosis, their coronary flow reserve (CFR) and microvascular resistance reserve (MRR) are already significantly impaired. This suggests that microcirculation damage precedes obvious cardiac structural alterations and is a crucial characteristic of early myocardial lesions in diabetes (22). However, few studies have simultaneously incorporated both microcirculatory function status and long-term metabolic markers to assess the risk of post-procedural MACCEs in patients with STEMI. Therefore, this multicenter retrospective cohort study aimed to investigate the combined prognostic value of post-PCI AMR and HbA1c levels for MACCEs in patients with STEMI and T2DM, and to explore their potential for improving risk stratification in this high-risk population.

## 2 Methods

### 2.1 Study design

**Figure 1:**
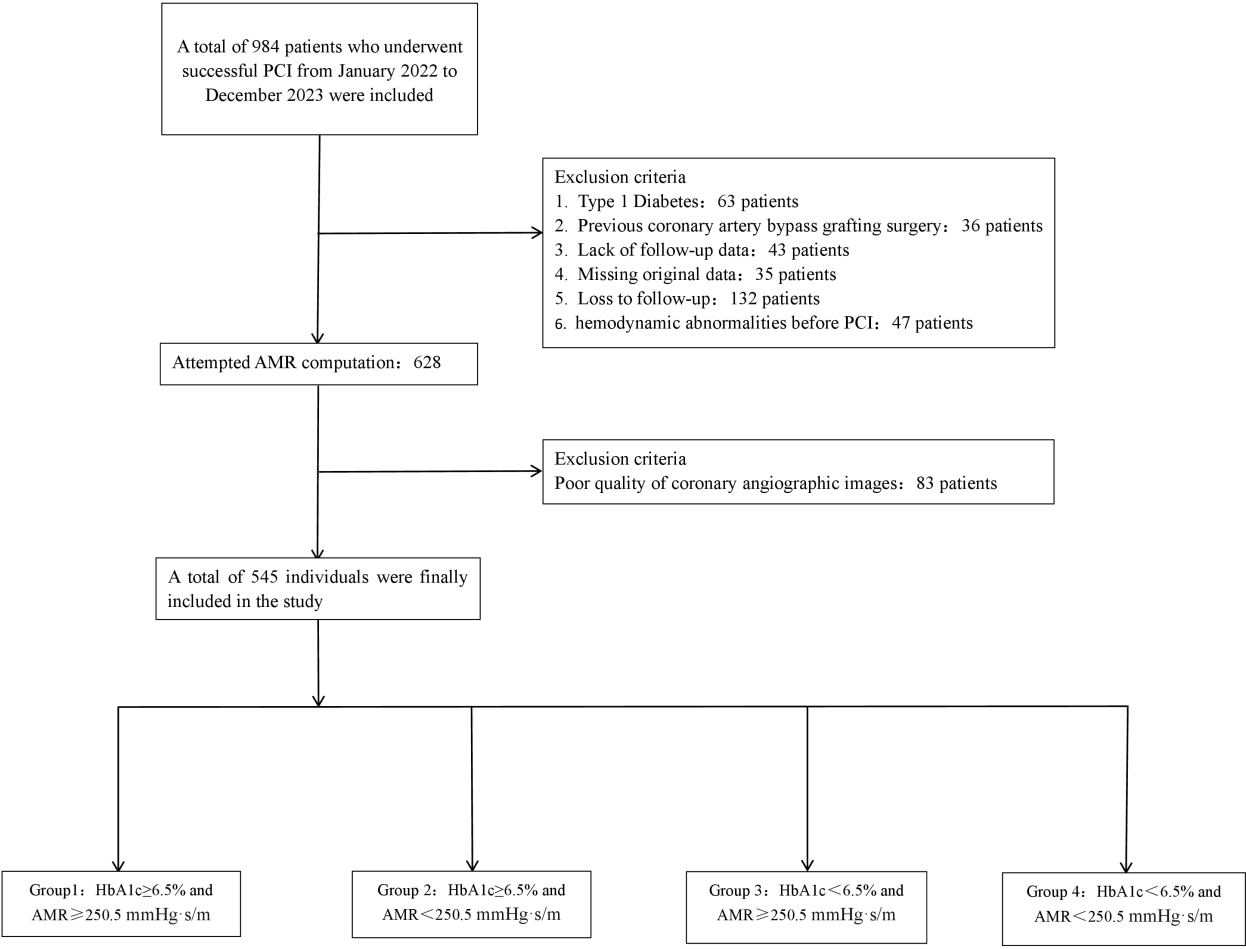
Patient screening flowchart. 984 patients who underwent PCI were screened during the period from January 2022, to December 2023. The study cohort comprised a total of 545 patients, who were included for further analysis. According to the HbA1c and AMR, the patients were categorized into four distinct groups. PCI, percutaneous coronary intervention; AMR, angio-based microvascular resistance; HbA1c: hemoglobin Alc.

This multicenter, retrospective observational study included a total of 984 patients diagnosed with STEMI in the emergency departments of The First Affiliated Hospital of Bengbu Medical University and The First People’s Hospital of Suzhou City between January 2022 and December 2023. According to the predefined exclusion criteria, 439 patients were excluded: 63 with type 1 diabetes mellitus, 36 with a history of coronary artery bypass grafting, 43 without follow-up data, 35 with missing original data, 132 lost to follow-up, 47 with hemodynamic abnormalities before PCI, and 83 with poor-quality coronary angiographic images that were not suitable for reliable QFR/AMR analysis. All exclusions were strictly carried out before group assignment. Ultimately, 545 patients were included in the analysis. Only patients with complete data for covariates and primary outcomes were included in the case analysis. The patient screening and exclusion process is detailed in **Figure 1**. The study protocol was approved by the ethics committees of The First Affiliated Hospital of Bengbu Medical University (Approval No. 2023KY046) and The Suzhou First People’s Hospital (Approval No. SZYYLLky2024016). The study followed the Declaration of Helsinki and applicable medical ethics guidelines.

### 2.2 Study population

Inclusion Criteria: Participants must be at least 18 years of age and have a confirmed diagnosis of STEMI. All patients must undergo PCI. According to the 2025 American College of Cardiology Guidelines for the Management of Patients with Acute Coronary Syndrome, STEMI is defined as the complete occlusion of a coronary artery resulting from thrombus formation over an atherosclerotic plaque, leading to an interruption in blood flow (23). According to the guidelines issued by the American Diabetes Association (ADA), the diagnostic criteria for T2DM are defined as follows: a fasting plasma glucose (FPG) level of ≥7.0 mmol/L, a 2-hour plasma glucose level of ≥11.1 mmol/L after an oral glucose tolerance test, a glycated hemoglobin (HbA1c) level of ≥6.5% (24). Successful PCI recanalization is defined by angiographic confirmation of residual stenosis of less than 30% and the restoration of coronary blood flow to a thrombolysis in myocardial infarction (TIMI) grade 3 level (25).

The exclusion criteria were as follows:

1. Patients diagnosed with Type 1 diabetes mellitus;
2. Individuals who had previously undergone coronary artery bypass grafting (CABG);
3. Participants who were lost to follow-up or had incomplete follow-up data;
4. Patients exhibiting significant hemodynamic instability prior to PCI;
5. Coronary angiography (CAG) images that were unsuitable for analysis due to factors such as blurring, nonstandard data formats, inadequate visualization, substantial vascular overlap, imaging artifacts, or cases in which only a single post-PCI angiographic image was available.

### 2.3 Laboratory examination

Blood samples were collected from STEMI patients immediately before PCI. HbA1c levels were measured using the fully automated Tosoh HLC-723G8 analyzer (Tokyo, Japan).

### 2.4 Measurement of AMR

The PCI procedure was performed by experienced interventional cardiologists in the operating room. Coronary angiographic images for AMR analysis were obtained only after successful recanalization of occluded vessels. Following successful recanalization of the target vessel, AMR analysis was performed using AngioPlus Gallery II software (Pulse Medical Technology Inc., Shanghai, China). The methodology is described in **Supplementary Material 1**. Research shows that the AMR calculation method has been subjected to inter-observer reproducibility analysis, and the results indicate a high level of consistency (ICC > 0.9) (25).

### 2.5 Outcome and follow-up

All patients received routine follow-up assessments via telephone interviews or outpatient clinic visits. The primary endpoint of this study was MACCEs, which were defined as a composite of all-cause mortality, hospital readmission for heart failure (HF), stroke (hemorrhagic or ischemic), any myocardial infarction, readmission for angina, and any revascularization during the follow - up period. In time - to - event analyses, only the first - occurring MACCE for each patient was taken into account. All-cause mortality is defined as the occurrence of death due to any cause during the follow - up period, encompassing sudden cardiac death (SCD) and non - cardiac death. SCD is defined as an unexpected death due to cardiac dysfunction, occurring typically within a short time period following the onset of symptoms and not attributable to any identifiable non-cardiac cause (26).

### 2.6 Statistical analysis

Statistical analyses were performed using SPSS version 27.0 (IBM SPSS Statistics, Chicago, IL, USA) and R version 4.4.2 (The R Foundation for Statistical Computing). The median and interquartile range (IQR) were reported. Continuous variables were expressed as median and IQR and compared between two independent groups using the Mann–Whitney U test. Categorical variables were expressed as counts and percentages and compared using the chi-square test or Fisher’s exact test, as appropriate. Baseline characteristics were compared descriptively within AMR strata, namely Group 1 versus Group 2 among patients with AMR ≥250.5 mmHg·s/m and Group 3 versus Group 4 among patients with AMR <250.5 mmHg·s/m. Baseline comparisons were performed for descriptive purposes only and were not intended for confirmatory hypothesis testing. Therefore, P values in **Table 1** were interpreted descriptively and were not used to define statistically significant baseline imbalance. For multiple pairwise comparisons of MACCE risk across the four risk groups, the Holm-Bonferroni method was applied to adjust for multiple comparisons. Both unadjusted and adjusted P values were reported where appropriate. To account for the imbalance of baseline covariates between groups, the inverse probability of treatment weighting (IPTW) method was employed (**Supplementary Material 2**). Kaplan-Meier curves were used to assess survival differences among the groups. Given the relatively limited quantity of MACCEs (n = 52), we employed LASSO regression (10 - fold cross - validation) to carry out a preliminary screening of candidate variables. Simultaneously, 100 bootstrap resampling procedures were performed to evaluate stability, and the frequency of each variable being selected in LASSO (lambda.min) was recorded. Ultimately, the variables selected in ≥ 50% of the bootstrap samples were identified. and the Log-rank test compared survival across the four groups. Hazard ratios (HR) with 95% confidence intervals (CI) were calculated using a multivariable Cox proportional hazards model to evaluate the relative risk of primary outcomes. We used a Cox proportional hazards model with restricted cubic splines (four knots) to generate HR curves. All analyses used two-tailed tests with p < 0.05 considered statistically significant. We assessed the proportional hazards assumption of the Cox proportional hazards model using Schoenfeld residuals (**eTable 1** in **Supplementary Material 3**). A global test and covariate-specific tests were performed; P values > 0.05 for both the overall test and individual covariates indicated that the proportional hazards assumption holds.

**Table 1:** Baseline characteristics.

Characteristics	AMR≥250.5 mmHg·s/m		*P	AMR<250.5 mmHg·s/m		^†^P
	HbA1c≥6.5% (n=74, Group 1)	HbA1c<6.5% (n=97, Group 2)		HbA1c≥6.5% (n=101, Group 3)	HbA1c<6.5% (n=273, Group 4)	
Study population						
Age, years	(58.00, 75.75)66.00	(53.00, 74.00)61.00	0.235	(56.00, 75.00)67.00	(54.00, 74.00)64.00	0.492
Male, n (%)	24 (32.43%)	19 (19.59%)	0.041	50 (49.50%)	132 (48.35%)	0.321
Cardiovascular risk factors						
Hypertension, n (%)	66 (89.19%)	91 (93.81%)	0.311	65 (64.36%)	76 (27.84%)	<0.001
Type 2 DM, n (%)	74 (100%)	3 (3.09%)	0.004	101 (100%)	14 (5.12%)	0.013
HbA1c (%)	(6.80, 8.40)7.30	(4.90, 5.70)5.20	0.015	(6.60, 7.20)6.90	(4.80, 5.70)5.20	0.032
Hyperlipemia, n (%)	43 (58.11%)	55 (56.70%)	0.889	26 (25.74%)	89 (32.60%)	0.358
Stroke, n (%)	15 (20.27%)	11 (11.34%)	0.06	30 (29.70%)	31 (11.36%)	<0.001
Smoking, n (%)	35 (47.30%)	17 (17.53%)	<0.001	57 (56.44%)	54 (19.78%)	<0.001
Previous CHD, n (%)	3 (4.05%)	4 (4.12%)	0.828	6 (5.94%)	21 (7.69%)	0.490
Previous PCI, n (%)	5 (6.76%)	4 (4.12%)	0.362	7 (6.93%)	16 (5.86%)	0.539
Pain-to-balloon time, min	(185.00,419.00) 296.00	(139.00,380.00) 215.00	0.003	(143.00,460.00) 266.00	(128.00,425.00) 255.00	0.880
Laboratory index						
cTnI, ng/l	(0.24, 13.40)1.53	(0.02, 5.00)0.24	0.004	(0.17, 12.40)3.86	(0.09, 9.65)1.63	0.532
NT‐proBNP, pg/mL	(103.97, 1199.50) 473.95	(66.43, 705.00)220.10	0.031	(85.00, 861.00)269.00	(79.00, 931.00)239.00	0.498
Creatinine, umol/l	(54.25, 83.75)70.50	(55.00, 77.00)65.00	0.312	(57.00, 81.00)67.00	(56.00, 80.00)68.00	0.575
CK/CKMB	(5.84, 11.17)8.10	(5.80, 9.30)7.10	0.374	(5.30, 9.56)7.43	(5.42, 9.88)7.59	0.931
TC-C, mmol/l	(3.81, 5.78)4.71	(3.96, 5.75)4.84	0.235	(3.76, 5.33)4.49	(3.77, 5.38)4.54	0.873
TG, mmol/l	(0.98, 2.31)1.62	(1.24, 2.74)1.78	0.215	(1.00, 2.27)1.44	(0.97, 1.98)1.43	0.829
HDL-C, mmol/l	(0.90, 1.28)1.07	(0.89, 1.22)1.02	0.280	(0.87, 1.32)1.06	(0.89, 1.26)1.04	0.681
LDL-C, mmol/l	(2.13, 3.07)2.42	(1.96, 3.59)2.92	0.036	(2.23, 3.35)2.65	(2.25, 3.33)2.75	0.931
Neutrophil, (10^9)	(5.97, 10.30)8.03	(5.16, 9.04)7.23	0.075	(5.72, 9.59)7.62	(5.50, 9.81)7.37	0.880
Monocyte, (10^9)	(0.29, 0.71)0.46	(0.40, 0.70)0.49	0.957	(0.31, 0.62)0.45	(0.41, 0.71)0.55	<0.001
Platelet, (10^9)	(182.75, 266.75)229.50	(175.00, 274.00) 220.00	0.903	(172.00, 266.00)224.00	(178.00, 272.00) 226.00	0.983
Lymphocyte, (10^9)	(0.96, 1.87)1.35	(1.21, 2.24)1.66	0.038	(0.82, 1.70)1.25	(1.07, 2.32)1.54	0.002
Aspirin, n (%)	71 (95.95%)	94 (96.91%)	0.69	98 (97.03%)	271 (99.27%)	0.13
Ticagrelor, n (%)	35 (47.30%)	53 (54.64%)	0.555	74 (73.27%)	209 (76.56%)	0.268
Clopidogrel, n (%)	37 (50.00%)	42 (43.30%)	0.656	26 (25.74%)	66 (24.18%)	0.487
Statins, n (%)	70 (94.59%)	95 (97.94%)	0.229	98 (97.03%)	268 (98.17%)	0.689
ACEI/ARB, n (%)	43 (58.11%)	55 (56.70%)	0.882	35 (34.65%)	107 (39.19%)	0.544
Beta‐blocker, n (%)	60 (81.08%)	82 (84.54%)	0.844	73 (72.28%)	209 (76.56%)	0.339
ARNi, n (%)	14 (18.92%)	17 (17.53%)	0.850	21 (20.79%)	83 (30.40%)	0.064
SGLT2i, n (%)	12 (16.22%)	13 (13.40%)	0.680	3 (2.97%)	16 (5.86%)	0.420
Spironolactone, n (%)	37 (50.00%)	47 (48.45%)	1	31 (30.69%)	97 (35.53%)	0.531
Furosemide, n (%)	33 (44.59%)	44 (45.36%)	0.767	26 (25.74%)	82 (30.04%)	0.599
Infarct‐related artery						
LAD, n (%)	38 (51.35%)	40 (41.24)	0.575	43 (42.57)	165 (60.44)	0.668
LCX, n (%)	12 (16.22%)	14 (14.43)	0.643	23 (22.77)	26 (9.52)	0.125
RCA, n (%)	24 (32.43%)	43 (44.33)	0.344	35 (34.65)	82 (30.04)	0.547
Multivessel disease						
1, n (%)	16 (21.62)	34 (35.05)	0.677	31 (30.69)	94 (34.43)	0.472
2, n (%)	40 (54.05)	41 (42.27)	0.231	54 (53.47)	140 (51.28)	0.421
3, n (%)	18 (24.33)	22 (22.68)	0.644	16 (15.84)	39 (14.29)	0.437
TIMI Flow Grade (initial)						
0	55 (74.32)	69 (71.13)	0.488	76 (75.25)	206 (75.46)	0.577
1	17 (22.97)	18 (18.56)	0.237	19 (18.81)	51 (18.68)	0.453
2	2 (2.71)	10 (10.31)	0.122	6 (5.94)	16 (5.86)	0.875
3	0 (0)	0 (0)	> 0.999	0 (0)	0 (0)	> 0.999
TIMI Flow Grade (post)						
0	0 (0)	0 (0)	> 0.999	0 (0)	0 (0)	> 0.999
1	0 (0)	0 (0)	> 0.999	0 (0)	0 (0)	> 0.999
2	0 (0)	0 (0)	> 0.999	0 (0)	0 (0)	> 0.999
3	74 (100)	97 (100)	> 0.999	101 (100)	273 (100)	> 0.999
QFR	(0.90, 0.98)0.94	(0.87, 0.96)0.91	0.138	(0.92, 0.98)0.97	(0.84, 0.97)0.92	<0.001
△QFR	(0.01, 0.09)0.05	(0.03, 0.12)0.07	0.374	(0.01, 0.07)0.03	(0.02, 0.13)0.06	<0.001
AMR	(262.00, 300.00)276.00	(262.00, 284.00)274.0	> 0.999	(213.00, 238.00) 230.00	(197.00, 234.00) 222.00	0.804
MACCEs, n (%)	18 (24.32%)	13 (13.40%)	0.045	10 (9.90%)	11 (4.03%)	0.313

AMR, angiography-derived microvascular resistance; HbA1c, hemoglobin A1c; DM, diabetes mellitus; CHD, Coronary Heart Disease; PCI, percutaneous coronary intervention; cTnI, cardiac troponin I; NT-proBNP, N-terminal pro b-type natriuretic peptide; CK/CKMB,creatine kinase/creatine kinase-MB; TC-C, total cholesterol; TG, triglycerides; HDL-C, high-density lipoprotein cholesterol; LDL-C, low-density lipoprotein cholesterol; ACEI, angiotensin-converting enzyme inhibitor; ARB, angiotensin II receptor blocker; ARNi, angiotensin receptor-neprilysin inhibitor; SGLT2i, sodium-glucose cotransporter 2 inhibitor; LAD, left anterior descending artery; LCX, left circumflex artery; RCA, right coronary artery; QFR, quantitative flow ratio; MACCEs, major adverse cardiovascular and cerebrovascular events. *P value for comparison between Group 1 and Group 2 within the AMR≥250.5 mmHg·s/m stratum. ^†^P value for comparison between Group 3 and Group 4 within the AMR<250.5 mmHg·s/m stratum. P values in **Table 1** are presented for descriptive purposes only and were not adjusted for multiple comparisons.

Because this was a retrospective cohort study including all eligible patients during the study period, no formal *a priori* sample-size calculation was performed before patient inclusion. Therefore, the sample size was determined by the number of eligible patients available in the participating centers during the predefined study period, rather than by prospective power calculation.

## 3 Results

### 3.1 Study population

A preliminary evaluation was conducted on a cohort of 984 patients diagnosed with STEMI who underwent PCI between January 2022 and December 2023. A total of 439 patients were excluded for failure to meet the prespecified inclusion criteria. Ultimately, a total of 545 patients were included in the study and underwent further analysis. The diagnostic threshold for diabetes based on HbA1c is 6.5%. ROC curve analysis identified a data-derived exploratory AMR cut-off value of 250.5 mmHg·s/m, with a sensitivity of 90% and a specificity of 71% (**eFigure 1** in **Supplementary Material 2**). Bootstrap resampling supported the internal stability of this cut-off (**eFigure 2** in **Supplementary Material 2**); however, this threshold should be considered exploratory pending external validation. Patients were categorized into four groups based on different levels of HbA1c and AMR: (1) Group 1: HbA1c ≥ 6.5% and AMR ≥ 250.5 mmHg·s/m, n = 74; (2) Group 2: HbA1c < 6.5% and AMR ≥ 250.5 mmHg·s/m, n = 97; (3) Group 3: HbA1c ≥ 6.5% and AMR < 250.5 mmHg·s/m, n = 101; (4) Group 4: HbA1c < 6.5% and AMR < 250.5 mmHg·s/m, n = 273 (**Figure 1**).

### 3.2 Baseline characteristics

Baseline characteristics across the four groups are presented in **Table 1**. In the subgroup with AMR ≥ 250.5 mmHg·s/m, Group 1 was distinguished by a greater proportion of females, higher HbA1c levels, more prevalent smoking, a longer interval from pain onset to balloon inflation, higher cTnI and NT-proBNP levels, lower LDL-c and lymphocyte counts, and a higher rate of MACCEs compared to Group 2. In the subgroup with AMR < 250.5 mmHg·s/m, Groups 3 and 4 exhibited descriptive disparities in hypertension, prior stroke, smoking, monocyte count, lymphocyte count, QFR, and residual QFR. These comparisons were incorporated to depict the distribution of clinical characteristics and should not be construed as conclusive statistical evidence. P values in **Table 1** are presented for descriptive purposes only and were not adjusted for multiple comparisons.

### 3.3 Univariate and multivariate Cox regression analysis for predicting MACCEs

**Figure 2:**
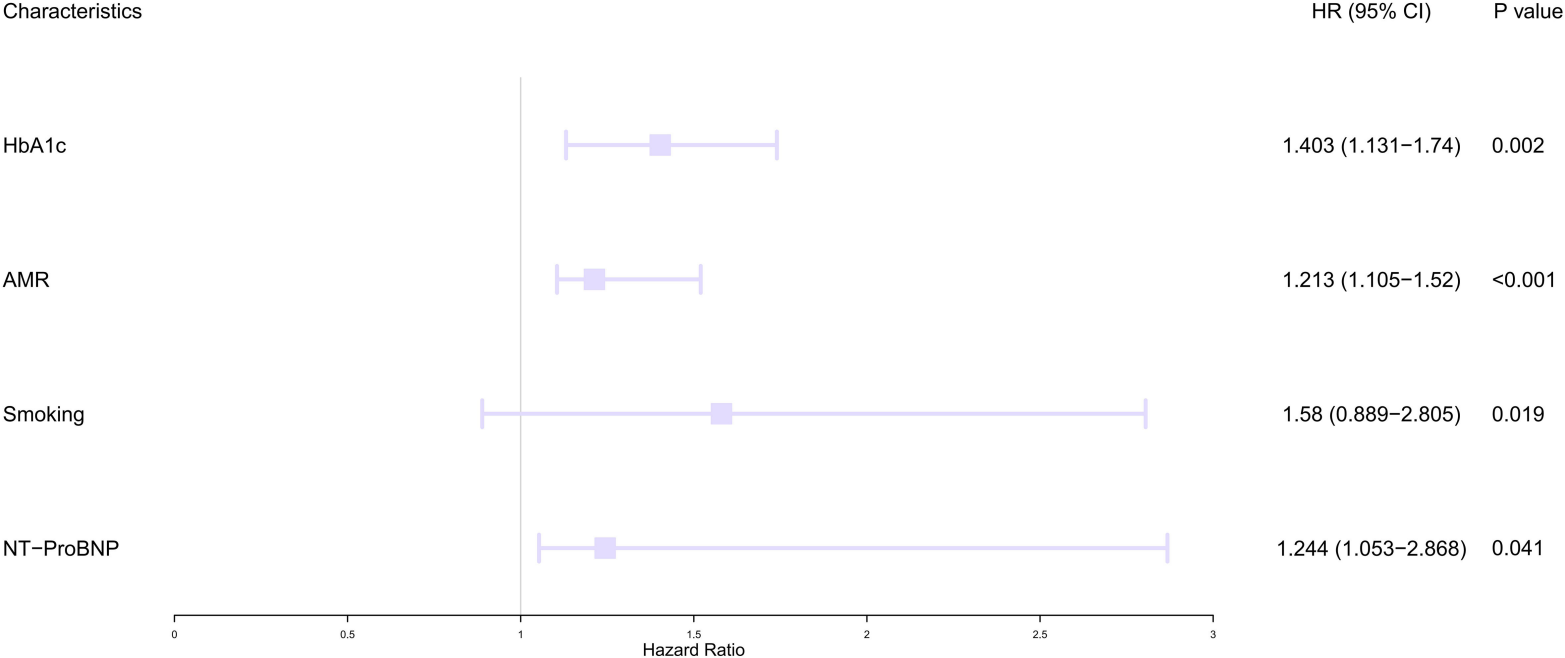
Multivariate Cox regression analysis for predicting MACCEs. AMR, angio-based microvascular resistance; HbA1c, hemoglobin Alc; NT-ProBNP, N-terminal pro-brain natriuretic peptide.

Univariate Cox regression analysis indicated that hypertension, T2DM, smoking, HbA1c levels, QFR, AMR, cTnI levels, and LDL-C levels were significantly associated with the occurrence of MACCEs (**eTable 2** in **Supplementary Material 3**). Due to the limited quantity of MACCEs events (n = 52), we initially employed LASSO regression in conjunction with Bootstrap stability assessment (100 resamplings) to screen the predictors. Ultimately, the four variables exhibiting the highest Bootstrap selection frequency (AMR, HbA1c, NT - proBNP, and smoking) were incorporated into the multivariate Cox proportional hazards model (**eFigure 3** in **Supplementary Material 2**). Overfitting was controlled with an EPV of approximately 13. The VIF values among multiple factors were simultaneously calculated, all of which were less than 5 (**eTable 3** in **Supplementary Material 3**). The findings indicate that HbA1c levels, AMR, and NT-proBNP levels are independently associated with the risk of MACCEs (**Figure 2**).

### 3.4 Univariate Cox regression analysis of MACCEs risk across different groups

During the one-year follow-up period, 52 first-occurring MACCEs were observed among the 545 included patients. The numbers of MACCEs in Groups 1–4 were 18/74, 13/97, 10/101, and 11/273, respectively, corresponding to incidences of 24.32%, 13.40%, 9.90%, and 4.03%. The incidence of MACCEs was markedly lower in Group 4 than in Groups 1, 2, and 3, with a statistically significant difference across the four groups (P < 0.0001). Univariate Cox regression analysis showed that HRs for MACCEs were significantly higher in the other groups compared to group 4 (**Table 2**). Group 1 had a 6.903-fold higher risk of MACCEs than the reference group (95% CI: 3.260–14.618, p < 0.001). Group 2 had a 3.509-fold increased risk (95% CI: 1.572–7.832, p = 0.002), and Group 3 had a 2.540-fold higher risk (95% CI: 1.079–5.981, p = 0.033). After Holm-Bonferroni correction for multiple pairwise comparisons, the increased risk of MACCEs remained statistically significant in Groups 1, 2, and 3 compared with Group 4.

**Table 2:** Comparison of MACEs risk among groups based on the Cox regression model.

Group (n,events/subjects)	HR	95%CI	Unadjusted P value	Holm-adjusted P value
Group 4 (11/273)	reference			
Group 1 (18/74)	6.903	3.260-14.618	<0.001	<0.003
Group 2 (13/97)	3.509	1.572-7.832	0.002	0.004
Group 3 (10/101)	2.540	1.079-5.981	0.033	0.033

HR, hazard ratios. P values were adjusted for multiple pairwise comparisons using the Holm-Bonferroni method.

### 3.5 Kaplan-Meier survival analysis

**Figure 3:**
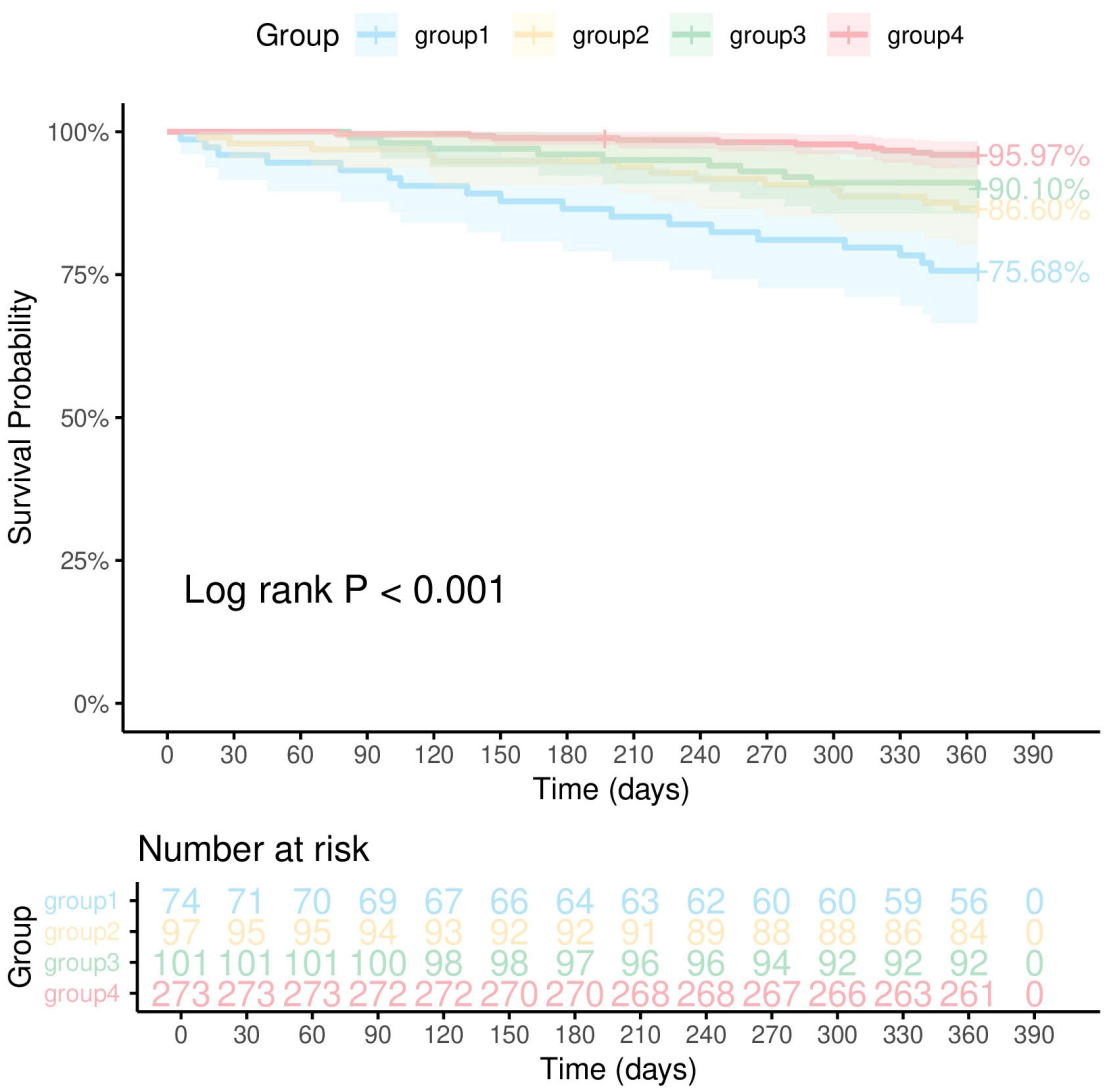
Survival curves of MACCEs in patients at different grouping levels.

During the one - year follow - up period, a total of 52 patients across the four study groups encountered MACCEs (**eTable 4** in **Supplementary Material 3**). The Kaplan-Meier analysis revealed a statistically significant difference in the incidence of MACCEs across the four groups (p < 0.001) (**Figure 3**). After adjusting for potential confounders using inverse probability of treatment weighting (IPTW), significant survival differences remained across the four patient groups (**eFigure 4** in **Supplementary Material 2**).

### 3.6 Analysis of the RCS curve

**Figure 4:**
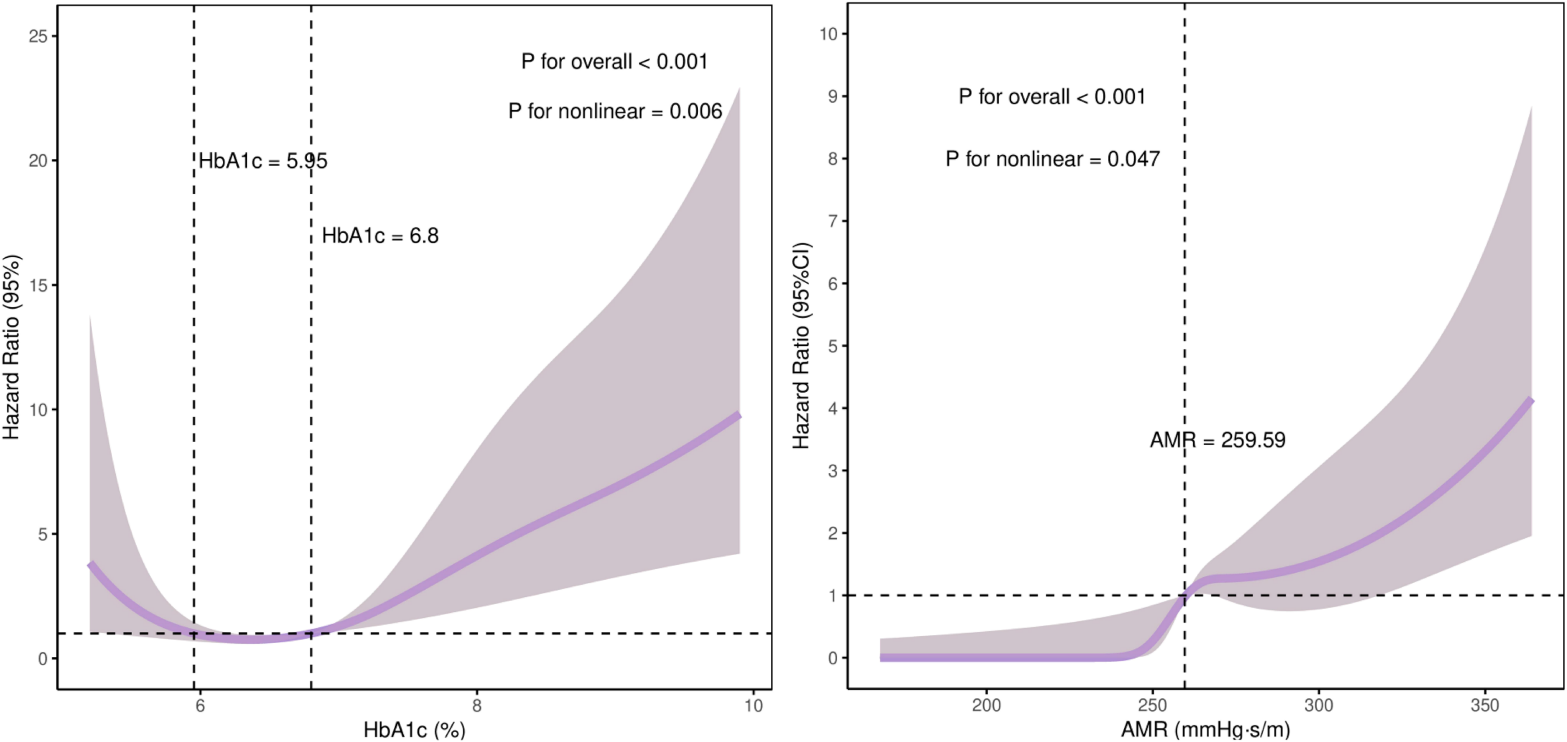
RCS analysis. AMR, angio-based microvascular resistance; HbA1c, hemoglobin Alc.

A nonlinear relationship was observed between MACCEs and both AMR and HbA1c values in both groups (**eFigure 5** in **Supplementary Material 2**). As determined by RCS analysis, the hazard ratio (HR) was greater than 1 when the HbA1c level was either below 5.95% or above 6.8% (P for overall < 0.001, P for nonlinear = 0.006). When the AMR reached 259.59 mmHg·s/m, the HR exceeded 1(P for overall < 0.001, P for nonlinear = 0.047) (**Figure 4**).

### 3.7 Relationship between HbA1c and AMR

To investigate the association between HbA1c and AMR, scatter plots were constructed to illustrate the correlation across three distinct cohorts: individuals with T2DM, those without T2DM, and the overall study population (**eFigure 6** in **Supplementary Material 2** and **eTable 5** in **Supplementary Material 3**). In the non-T2DM group, no statistically significant effect of HbA1c on AMR was observed (adjusted R² = 0.002, P = 0.187). In contrast, among individuals with T2DM, HbA1c demonstrated a significant association with AMR (adjusted R² = 0.267, P < 0.001). When analyzing the entire study population, HbA1c remained significantly associated with AMR (adjusted R² = 0.117, P < 0.001).

## 4 Discussion

The primary objective of this study was to evaluate the prognostic significance of combining HbA1c with AMR for assessing CMD in patients with STEMI following PCI. The principal findings of this study are as follows: (1) Significant differences in prognosis were observed across different patient groups, particularly among individuals with HbA1c ≥ 6.5% and AMR ≥ 250.5 mmHg·s/m, who exhibited poorer clinical outcomes. (2) A statistically significant association was identified between HbA1c levels and AMR among patients diagnosed with T2DM. (3) A nonlinear relationship was found between HbA1c, AMR, and the incidence of MACCEs. Specifically, when AMR exceeded 259.59 mmHg·s/m, a marked increase in the risk of MACCEs was observed for patients with HbA1c levels either below 5.95% or above 6.8%.

Studies have consistently demonstrated that patients diagnosed with T2DM are at a significantly higher risk of long-term mortality following an episode STEMI (8, 9). The pathophysiological mechanisms underlying the association between hyperglycemia and adverse clinical outcomes are heterogeneous and involve multiple interrelated pathways. Chronic hyperglycemia is a key contributor to both macrovascular and microvascular endothelial dysfunction (27). This pathophysiological process arises from the synergistic interaction among hyperglycemia, oxidative stress, and chronic low-grade inflammation, leading to progressive endothelial dysfunction. Elevated blood glucose levels can directly compromise the activity of antioxidant enzymes, such as superoxide dismutase (SOD), thereby exacerbating oxidative stress (28). Moreover, advanced glycation end products (AGEs) contribute to endothelial dysfunction by reducing both the stability and half-life of endothelial nitric oxide synthase (eNOS) mRNA (27).

In individuals with T2DM, elevated HbA1c concentrations reflect suboptimal long-term glycemic control (24). Multiple studies have reported an independent association between elevated HbA1c concentrations and increased long-term all-cause mortality in adults with T2DM (29). Elevated AMR is associated with impaired restoration of coronary microcirculatory perfusion. Even when major vessels are successfully recanalized, persistent microcirculatory hypoperfusion often continues to impair myocardial function. Vessels exhibiting elevated microcirculatory resistance are often associated with pronounced inflammatory responses. Such inflammation may not only contribute to vascular endothelial injury and thrombosis but also further impair microcirculatory function (30). Persistent disturbances in the microcirculation not only accelerate cardiac remodeling but also play a significant role in the progression of ventricular hypertrophy and myocardial fibrosis.

At present, there is no definitive consensus on the exact role of AMR in the diagnosis of CMD. Zhang et al. identified a critical threshold of 236 mmHg·s/m for AMR in predicting CMD (31). Luo et al. reported that patients with AMR ≥250 mmHg·s/m had a significantly higher risk of MACCEs (32). However, the mean follow-up duration in this study was 30 days. Onishi et al. reported that an AMR ≥250 mmHg·s/m in at least one coronary artery may serve as a potential diagnostic threshold for CMD (33). Qian et al. identified 255 mmHg·s/m as the optimal AMR threshold using the maximal selected log-rank statistic method (34). The AMR cut-off value of 250.5 mmHg·s/m identified in this study is close to thresholds reported in previous studies. Nevertheless, this value was derived from ROC analysis within the present cohort and was supported only by internal bootstrap resampling. Therefore, it should be regarded as an exploratory, data-driven threshold rather than a definitive clinical decision boundary. External validation in independent and prospective cohorts is required before this cut-off can be recommended for routine clinical risk stratification.

In this study, two clinically pertinent potential confounders were identified: (1) the anatomical and functional integrity of the revascularized coronary vasculature, assessed postoperatively using the TIMI flow grade; and (2) glycemic control during the post-discharge period. Multivariate Cox proportional hazards regression analysis revealed no statistically significant association between either variable and the risk of MACCEs (P > 0.05). The high success rate of revascularization attenuates the discriminative capacity of this endpoint for risk stratification. Furthermore, Glycemic control following hospital discharge is a dynamic process; therefore, in-hospital glucose measurements obtained during acute admission may not reliably capture longitudinal glycemic patterns (35). In addition, infarct size is a well-established prognostic determinant. Owing to the retrospective design of this study, standardized quantification of infarct size was not available for all enrolled patients (36). In this study, SGLT2 inhibitor use was observed in only a limited proportion of participants in this study. Accumulating clinical evidence supports cardioprotective benefits of SGLT2 inhibitors in patients with acute myocardial infarction (37). Proposed mechanisms include attenuation of oxidative stress, inhibition of pro-fibrotic signaling pathways, and stabilization of coronary atherosclerotic plaques (37). Differential utilization of SGLT2 inhibitors across subgroups may have contributed to observed variations in clinical outcomes. This imbalance represents an important methodological constraint that should be considered. Future prospective studies should systematically collect comprehensive medication data and address this confounding factor through rigorous analytical strategies, thereby enabling a more robust evaluation of the independent predictive value of AMR.

This study indicates that the concurrent assessment of AMR and HbA1c may support postoperative risk stratification in patients with STEMI and T2DM. In the present cohort, patients with AMR ≥ 250.5 mmHg·s/m and HbA1c ≥ 6.5% represented the subgroup with the highest observed risk of MACCEs. However, this AMR threshold remains exploratory and should not be regarded as a definitive clinical decision boundary until validated in independent cohorts. In the present cohort, the overall incidence of MACCEs was 9.5% (52/545). Notably, patients with both elevated HbA1c (≥6.5%) and AMR (≥250.5 mmHg·s/m) had the highest incidence of MACCEs (24.3%, 18/74). For patients with elevated HbA1c levels, early initiation of glucose-lowering agents with robust, evidence-based cardiovascular benefits is recommended to improve glycemic control. In patients with elevated AMR, persistent myocardial microcirculatory dysfunction may remain clinically relevant despite successful epicardial coronary revascularization. Adjunctive pharmacologic strategies targeting microcirculatory improvement or enhanced anti-inflammatory modulation may be considered, pending further evidence from rigorously designed clinical studies. AMR can be derived noninvasively from standard coronary angiography images using validated computational methods. Meanwhile, HbA1c provides a stable, integrative biomarker reflecting average glycemia over the preceding 2–3 months. The combined utilization of the two exhibits the characteristics of simple operation, controllable cost, and high clinical accessibility, which facilitates the precise identification of postoperative risks and individualized intervention within the routine clinical pathway.

## 5 Limitations

This study has several limitations. First, retrospective studies are susceptible to selection bias, which limits the validity of causal inference. Although we adjusted for potential confounders, residual confounding from unmeasured or unknown variables remains possible. Thus, the observed associations between AMR, HbA1c, and MACCEs are correlational—not causal. Secondly, HbA1c reflects the average blood glucose concentration over the preceding 8–12 weeks and is comparatively less susceptible to acute physiological perturbations; however, transient elevations in HbA1c may occur under conditions of acute physiological stress even in the absence of* chronic hyperglycemia. Thirdly, some patients were excluded from the QFR analysis due to poor angiographic image quality, which may introduce selection bias. Moreover, the computation of functional indices derived from angiographic data is inherently dependent on the underlying physical models and the assumptions regarding boundary conditions implemented in the computational software. The computational model used in this study incorporates Murray’s law and the laminar flow assumption for hemodynamic simulation. However, these simplifying physical assumptions do not fully account for inter-individual variability in vascular geometry, blood rheology, and dynamic physiological conditions. Additionally, because this was a retrospective study and no formal *a priori* sample-size calculation was performed, the sample size was determined by the number of eligible patients available during the predefined study period. With 545 patients and 52 observed MACCEs, the study may have been underpowered to detect modest effect sizes or subgroup-specific associations. Larger prospective studies with predefined sample-size calculations are warranted to validate these findings. Finally, although a parsimonious modeling strategy was adopted to constrain the number of covariates in the multivariate analysis—thereby improving model generalizability and interpretability—the possibility of residual overfitting cannot be entirely ruled out. Therefore, this study should be interpreted as an exploratory analysis, and its primary findings warrant validation in a larger, prospective cohort study.

## 6 Conclusion

This study demonstrates that an elevated AMR following PCI independently predicts MACCEs in patients with STEMI and T2DM. Combined evaluation of AMR and HbA1c enhances risk stratification for MACCEs. Assessment of coronary microcirculatory function and sustained optimization of glycemic control may contribute to improved long-term clinical outcomes in this population.

## Data Availability

The original contributions presented in the study are included in the article/**Supplementary Material**. Further inquiries can be directed to the corresponding authors.
