# Supplementary material for: Elevated angiography-derived microvascular resistance and HbA1c levels jointly predict adverse outcomes in patients with diabetic STEMI: a multicenter retrospective cohort study
Source: Front Endocrinol (Lausanne). 2026 Jun 22;17:1756159. doi: 10.3389/fendo.2026.1756159 (PMC13333462; doi:10.3389/fendo.2026.1756159)
Supplement: Supplementary file 1 [file DataSheet1.docx]

**Supplementary material 1**

Shiyi Gao, Yu Wang, Jun Wang, et al. Elevated Angiography-Derived Microvascular Resistance and HbA1c Levels Jointly Predict Adverse Outcomes in Patients with Diabetic STEMI: A Multicenter Retrospective Cohort Study.

**Calculation of AMR**

Following reperfusion of the culprit vessel, the version AngioPlus Gallery II software (Pulse Medical Technology Inc., Shanghai, China) was used for offline single-view QFR and AMR analysis based on Murray’s law. The software initially conducts an automatic recognition of the lumen contours of the target vessel and its principal branches. In cases where the outcomes of the automatic recognition deviate notably from the actual vessel boundaries, upon magnifying the image to ensure a clear display of the vessel contours, the analyst manually rectifies the contours. Upon completion of manual correction, another colleague reviewed the correction results to verify that the contour tracing was substantially consistent with the vascular anatomical boundaries. This study did not perform a formal repeatability analysis of AMR measurements within the current cohort, nor did it conduct a statistical evaluation of intra - observer consistency and inter - observer consistency.


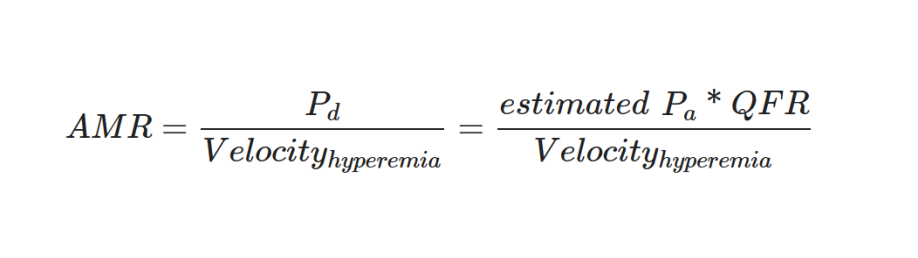


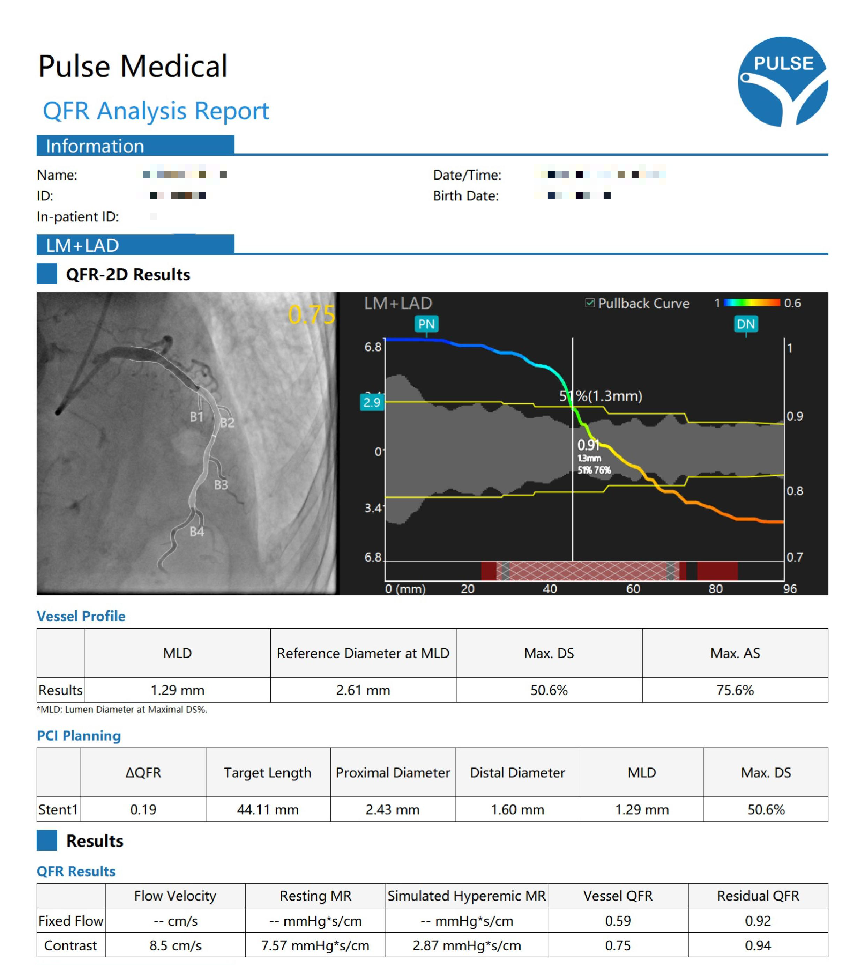


A 76-year-old female patient successfully underwent PCI in the culprit vessel, the LAD, and subsequently achieved hemodynamic stability. Immediately thereafter, quantitative assessments of the QFR and AMR were conducted. The results revealed a QFR value of 0.75 and an AMR value of 287 mmHg·s/m.
