# Supplementary material for: Elevated angiography-derived microvascular resistance and HbA1c levels jointly predict adverse outcomes in patients with diabetic STEMI: a multicenter retrospective cohort study
Source: Front Endocrinol (Lausanne). 2026 Jun 22;17:1756159. doi: 10.3389/fendo.2026.1756159 (PMC13333462; doi:10.3389/fendo.2026.1756159)
Supplement: Supplementary file 2 [file DataSheet2.docx]

**Supplementary material 2**

Shiyi Gao, Yu Wang, Jun Wang, et al. Elevated Angiography-Derived Microvascular Resistance and HbA1c Levels Jointly Predict Adverse Outcomes in Patients with Diabetic STEMI: A Multicenter Retrospective Cohort Study.

**eFigure 1:** The diagnostic efficacy of AMR for predicting MACCEs.

**eFigure 2:** Stability Validation of the Optimal AMR ROC Cutoff Using Bootstrap Resampling.

**eFigure 3:** Bootstrap LASSO Variable Selection Frequency.

**eFigure 4:** Outcomes after implementing the IPTW method adjustment (The description of the IPTW method is included).

**eFigure 5：**The relationship between various independent variables and regression residuals.

**eFigure 6:** Association between HbA1c and AMR.

This supplemental material has been provided by the authors to give readers additional information about their work.

**eFigure 1:** The diagnostic efficacy of AMR for predicting MACCEs.


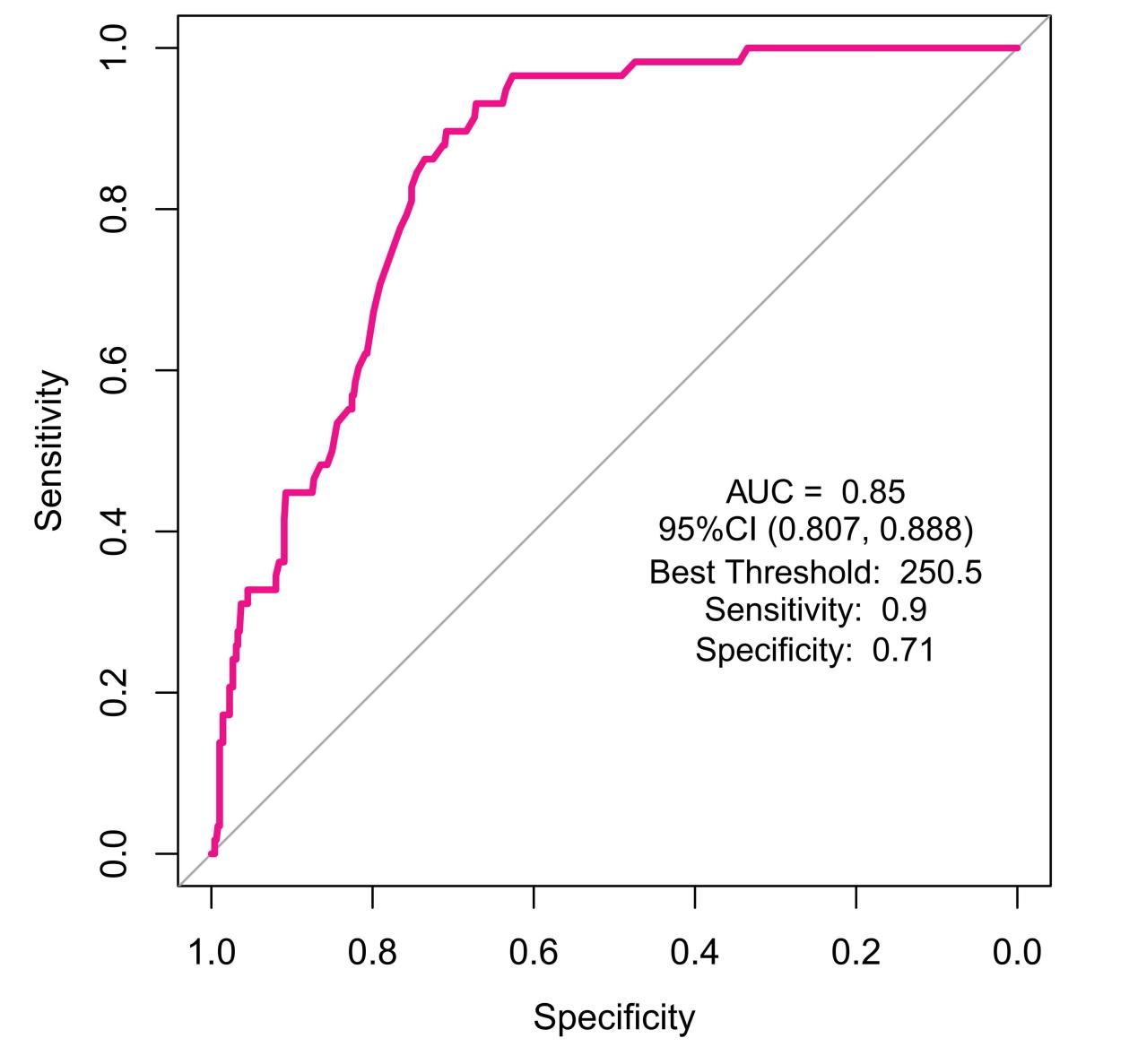


The diagnostic efficacy of AMR for predicting MACCEs. Best Cutoff: 250.5 mmHg·s/m, AUC= 0.85,sensitivity: 90% , specificity: 71%.

**eFigure 2:** Stability Validation of the Optimal AMR ROC Cutoff Using Bootstrap Resampling.

**
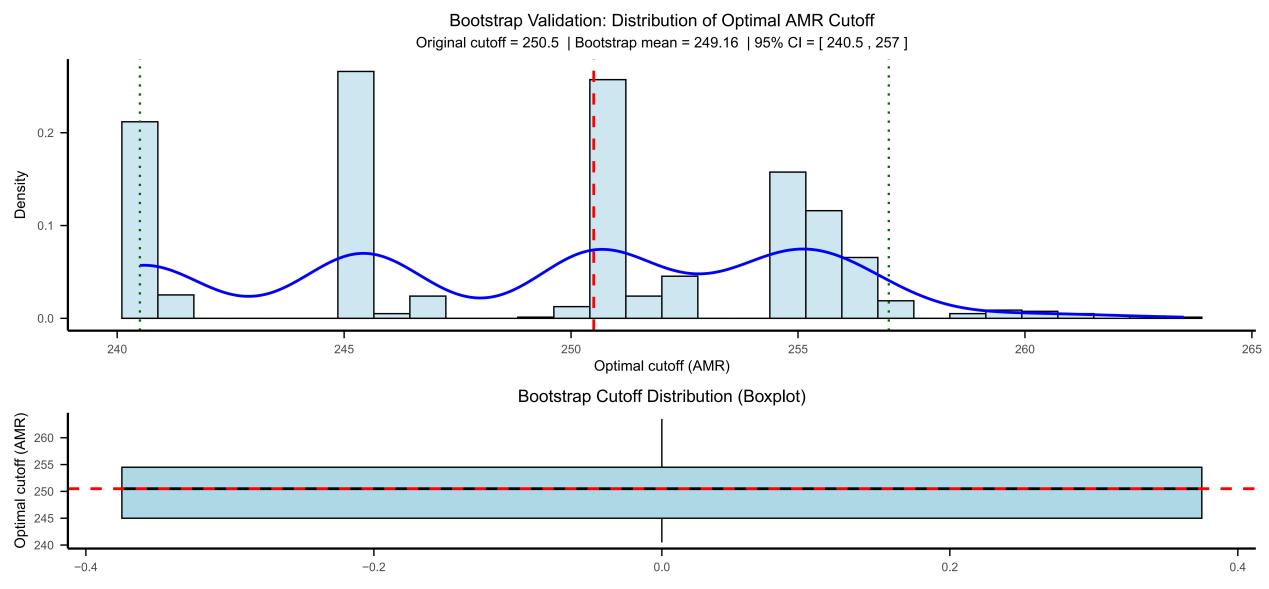
**

**eFigure 3:** Bootstrap LASSO Variable Selection Frequency.

**
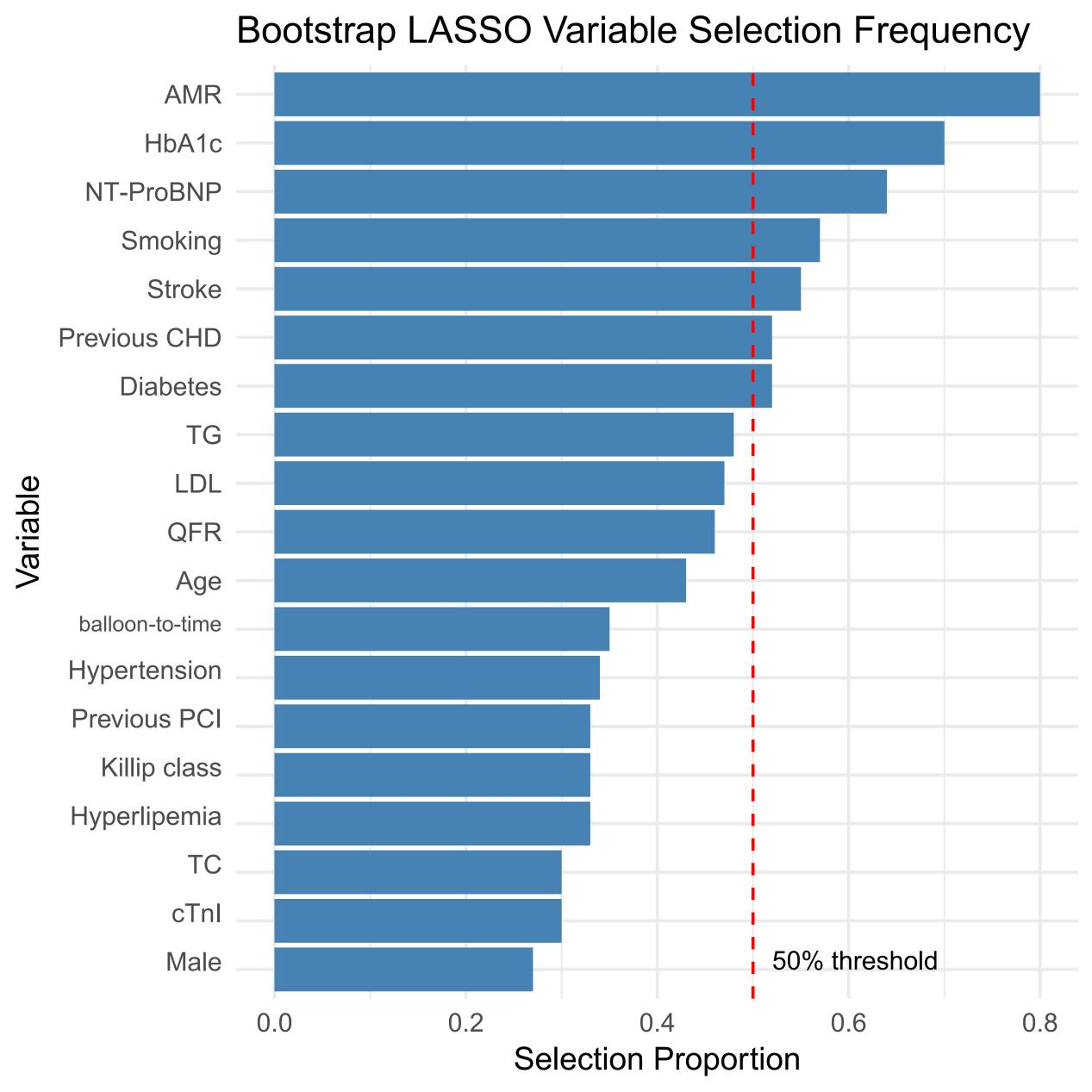
**

**eFigure 4:** Outcomes after implementing the IPTW method adjustment.


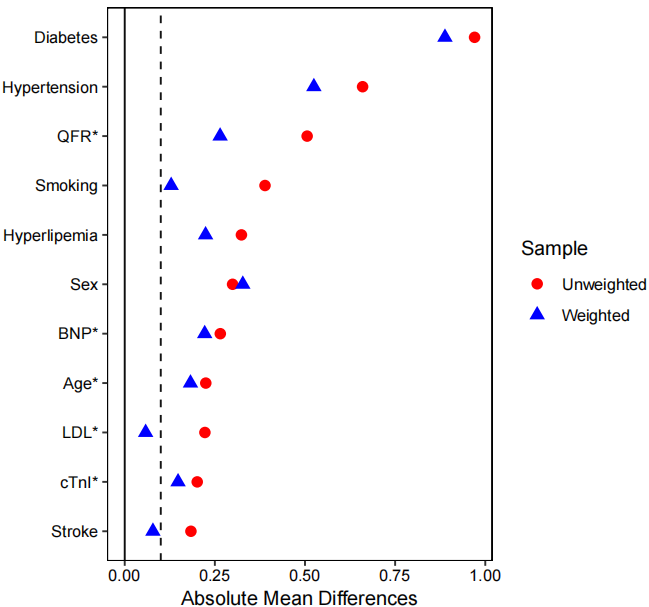

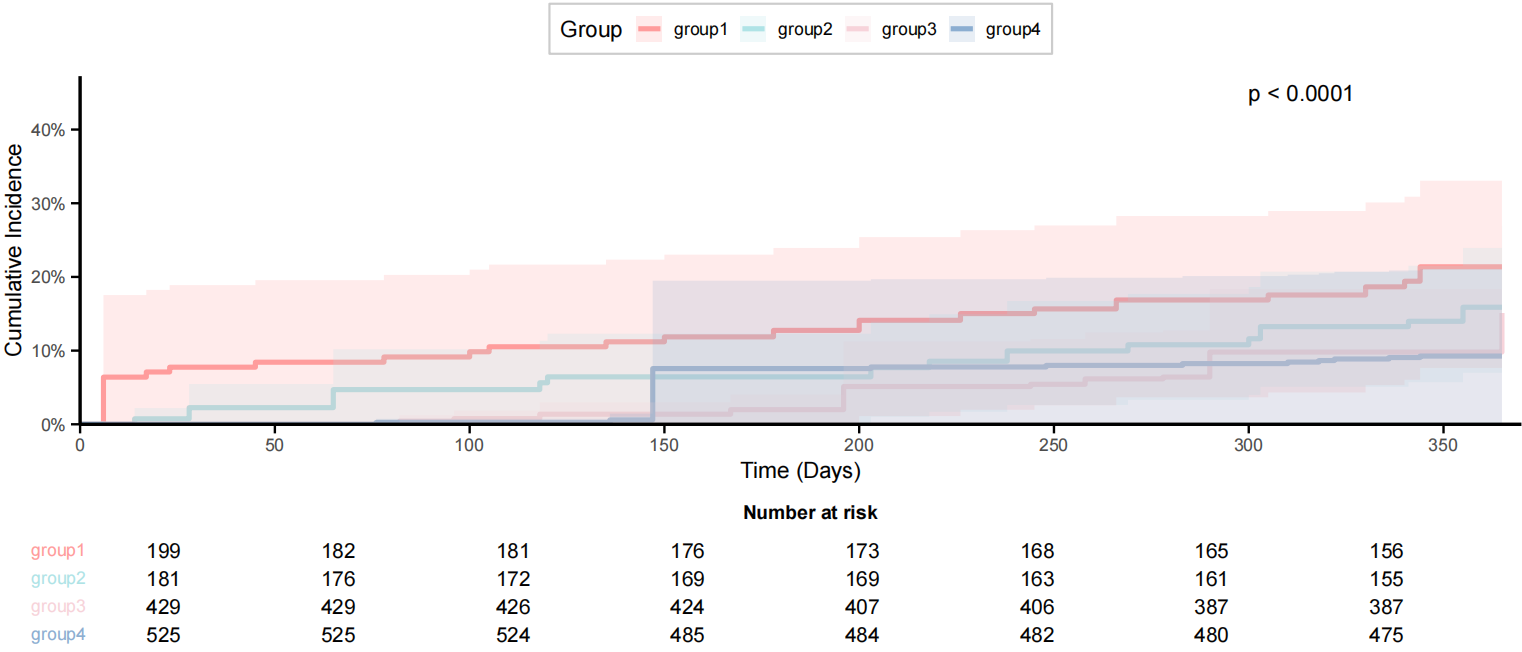


Following adjustment for potential confounding factors using inverse probability of treatment weighting , significant differences in survival outcomes persisted among the four patient groups (P＜0.0001).

To account for the imbalance of baseline covariates among groups, the IPTW method was employed. Initially, a multinomial logistic regression model was established, with the four - category grouping variable "Group" serving as the dependent variable. Age, gender, hypertension, diabetes, hyperlipidemia, history of stroke, smoking history, NT-ProBNP, cTnI, LDL, and QFR were included as covariates to estimate the propensity scores. The propensity scores were computed using the "ps" method in the "WeightIt" package of R software (for multi - class treatment variables). The target population was set as the average treatment effect (ATE), and the weighting formula was wi = 1/P(T = ti | Xi). Subsequent to weighting, the standardized mean difference was utilized to evaluate the balance of covariates. The "cobalt" package was employed to generate the love plot, with an absolute value of the standardized difference < 0.1 regarded as the acceptable criterion for group balance. For the limited number of missing values in the covariates, complete case analysis was adopted. Specifically, any individual with missing values was excluded prior to model fitting. Ultimately, the weighted Kaplan - Meier method was applied to estimate the cumulative incidence (1 - survival rate) of each group, and the log - rank test was used to compare the differences between groups. The weighted survival curves were obtained through the "survfit" function in the "survival" package, with the weights being the IPTW weights.

**eFigure 5：**The relationship between various independent variables and regression residuals. AMR, angio-based microvascular resistance; HbA1c: hemoglobin A1c.


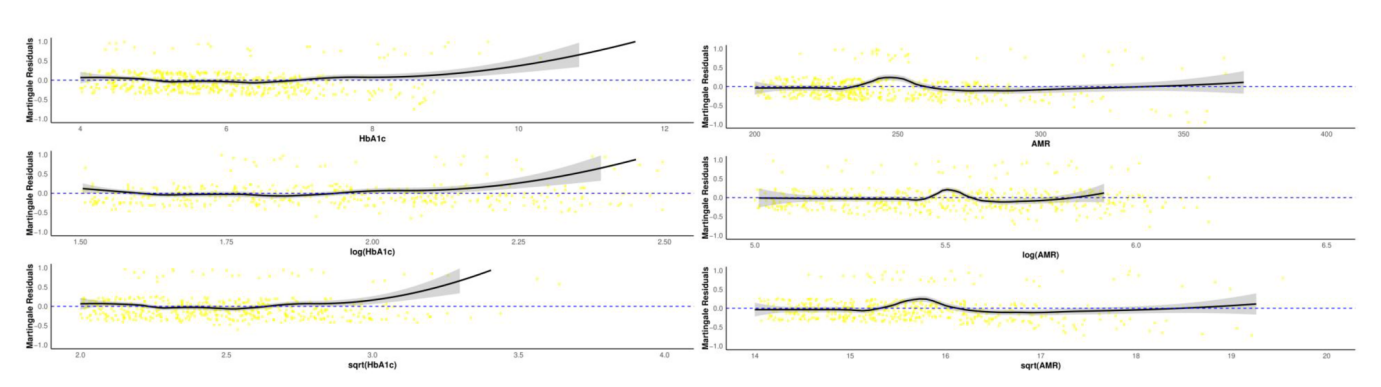


**eFigure 6:** Association between HbA1c and AMR.


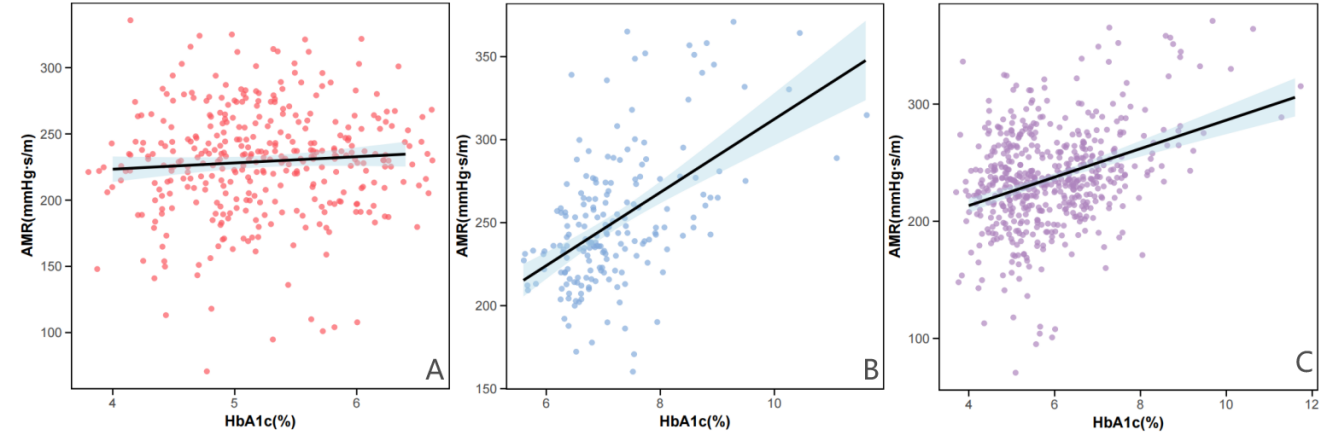


Scatter plots were constructed to illustrate the correlation across three distinct cohorts: individuals with T2DM (A), those without T2DM (B), and the overall study population (C). In the non-T2DM group, no statistically significant effect of HbA1c on AMR was observed (adjusted R² = 0.002, P = 0.187). In contrast, among individuals with T2DM, HbA1c demonstrated a significant association with AMR (adjusted R² = 0.267, P < 0.001). When analyzing the entire study population, HbA1c remained significantly associated with AMR (adjusted R² = 0.117, P < 0.001).
