# Supplementary material for: Elevated angiography-derived microvascular resistance and HbA1c levels jointly predict adverse outcomes in patients with diabetic STEMI: a multicenter retrospective cohort study
Source: Front Endocrinol (Lausanne). 2026 Jun 22;17:1756159. doi: 10.3389/fendo.2026.1756159 (PMC13333462; doi:10.3389/fendo.2026.1756159)
Supplement: Supplementary file 3 [file DataSheet3.docx]

**Supplementary material 3**

Shiyi Gao, Yu Wang, Jun Wang, et al. Elevated Angiography-Derived Microvascular Resistance and HbA1c Levels Jointly Predict Adverse Outcomes in Patients with Diabetic STEMI: A Multicenter Retrospective Cohort Study.

**eTable 1:** Schoenfeld residual test for the proportional hazards assumption of the Cox proportional hazards model.

**eTable 2:** Univariate Cox regression analysis for predicting MACCEs.

**eTable 3:** VIF calculation.

**eTable 4：**Outcomes.

**eTable 5:** Summary of the Association Between HbA1c and AMR Across Different Cohorts.

This supplemental material has been provided by the authors to give readers additional information about their work.

**eTable 1: Schoenfeld residual test for the proportional hazards assumption of the Cox proportional hazards model.**

| Variable | chisq | df | p |
| --- | --- | --- | --- |
| HbA1c | 1.67584952038088 | 1 | 0.19523663836566821 |
| AMR | 1.27354586946728 | 1 | 0.2593753534029883 |
| Smoking | 2.521833332 | 1 | 0.112279979035528 |
| BNP | 2.971895711 | 1 | 0.0847225195585043 |
| GLOBAL | 8.4431244332945 | 4 | 0.07664229854630894 |

**eTable 2: Univariate Cox regression analysis for predicting MACCEs.**

| Characteristics | HR | 95%CI | P value | Characteristics | HR | 95%CI | P value |
| --- | --- | --- | --- | --- | --- | --- | --- |
| Male | 0.686 | 0.384-1.225 | 0.203 | NT-ProBNP* | 1.361 | 1.193-3.133 | 0.042 |
| Hypertension* | 1.783 | 1.235-2.877 | 0.004 | Lymphocyte | 0.742 | 0.539-1.022 | 0.068 |
| Type 2 DM* | 3 | 1.724-5.221 | ＜0.001 | CTnI* | 1.234 | 1.123-2.548 | 0.048 |
| Hyperlipemia | 1.366 | 0.792-2.356 | 0.263 | QFR* | 0.857 | 0.623-0.973 | 0.044 |
| Stroke | 0.691 | 0.295-1.619 | 0.395 | AMR* | 1.120 | 1.013-1.568 | ＜0.001 |
| Smoking* | 2.313 | 1.342-3.985 | 0.003 | Pain-to-balloon time | 1.325 | 1.106-2.649 | 0.067 |
| HbA1c* | 1.749 | 1.463-2.090 | ＜0.001 | LDL-C* | 1.237 | 1.018-3.533 | 0.028 |

DM, diabetes mellitus; HbA1c, glycated Hemoglobin A1c; cTnI,cardiac troponin I; QFR, quantitative flow ratio; AMR,angio-derived microvascular resistance; LDL-C,low-density lipoprotein cholesterol.

**eTable 3: VIF calculation.**

| characteristics | tolerance | VIF |
| --- | --- | --- |
| HbA1c | 0.862 | 1.159 |
| AMR | 0.817 | 1.223 |
| Smoking | 0.817 | 1.081 |
| NT | 0.969 | 1.081 |

**eTable 4：Outcomes.**

|  | Total | Group1 | Group2 | Group3 | Group4 | P value |
| --- | --- | --- | --- | --- | --- | --- |
| MACCEs | 52 (9.54%) | 18 (24.32%) | 13 (13.40%) | 10 (9.90%) | 11 (4.03%) | <0.001 |
| All-cause mortality | 17 (3.12%) | 6 (8.11%) | 4 (4.12%) | 3 (2.97%) | 4 (1.47%) | <0.001 |
| Hospital readmission for heart failure | 29 (5.32%) | 9 (12.16%) | 6 (6.19%) | 6 (5.94%) | 8 (2.93%) | 0.027 |
| Any myocardial infarction | 9 (1.65%) | 4 (5.40%) | 0 (0.00%) | 1 (0.99%) | 4 (1.46%) | 0.074 |
| IRA myocardial infarction | 4 (0.73%) | 1 (1.35%) | 0 (0.00%) | 1 (0.99%) | 2 (0.73%) | 0.572 |
| Non-IRA myocardial infarction | 5 (0.92%) | 3 (4.05%) | 0 (0.00%) | 0 (0.00%) | 2 (0.73%) | 0.101 |
| Readmission for angina | 20 (3.67%) | 8 (10.81%) | 4 (4.12%) | 5 (4.95%) | 3 (1.10%) | 0.092 |
| Any revascularization | 15 (2.75%) | 5 (6.76%) | 3 (3.09%) | 2 (1.98%) | 5 (1.83%) | 0.081 |
| Stroke | 3 (0.55%) | 1 (1.35%) | 0 (0.00%) | 0 (0.00%) | 2 (0.73%) | 0.484 |

IRA,infarct-related artery.

**eTable 5:** Summary of the Association Between HbA1c and AMR Across Different Cohorts.

| Group | Adjusted R^2^ | P value |
| --- | --- | --- |
| Non-T2DM | 0.002 | 0.187 |
| T2DM | 0.267 | ＜0.001 |
| All | 0.117 | ＜0.001 |
